# Monitoring the health and healthcare provision for refugees in collective accommodation centres: Results of the population-based survey RESPOND

**DOI:** 10.25646/7863

**Published:** 2021-03-31

**Authors:** Louise Biddle, Maren Hintermeier, Amir Mohsenpour, Matthias Sand, Kayvan Bozorgmehr

**Affiliations:** 1 Section Health Equity Studies and Migration, Department of General Practice and Health Services Research, University Hospital Heidelberg; 2 AG Population Medicine and Health Services Research, School of Public Health, Bielefeld University; 3 GESIS Leibniz Institute for the Social Sciences, Mannheim

**Keywords:** HEALTH MONITORING, REFUGEES, SURVEY, ACCESS BARRIERS, QUALITY OF CARE

## Abstract

To date, the integration of refugees in German health surveys is insufficient. The survey RESPOND (Improving regional health system responses to the challenges of forced migration) aimed to collect valid epidemiological data on refugee health status and healthcare provision. The core elements of the survey consisted of a population-based sampling procedure in Baden-Württemberg, multilingual questionnaires and a face-to-face approach of recruitment and data collection in collective accommodation centres with multilingual field teams. In addition, data on the geographical locations of accommodation centres and their structural quality were obtained. The results indicate a high overall health burden. The prevalence of depression (44.3%) and anxiety symptoms (43.0%) was high. At the same time, high unmet needs were reported for primary (30.5%) and specialist (30.9%) care. Despite sufficient geographical accessibility of primary care services, frequent ambulatory care sensitive hospitalisations, i.e. hospitalisations that could potentially have been avoided through primary care (25.3%), as well as subjective deficits in the quality of care, suggest barriers to accessing healthcare services. Almost half of all refugees (45.3%) live in accommodation facilities of poor structural quality. Collecting valid data on the health situation of refugees is possible through a combination of targeted sampling, multilingual recruitment and survey instruments as well as personal recruitment. The presented approach could complement established procedures for conducting health surveys and be extended to other federal states.

## 1. Introduction

Due to experiences before, during and after flight, refugees (Info box 1) face specific health risks, which makes an efficient healthcare response after arrival in Germany crucial. International studies show that providing care for mental health issues, chronic diseases, serious infectious diseases as well as for pregnant women is particularly important [1].

Ensuring that refugees in Germany receive adequate healthcare is challenging. The legal norms of the Asylum Seekers Benefits Act (‘Asylbewerberleistungsgesetz’, AsylbLG) limit care to the ‘treatment of acute illnesses and pain conditions’ (§4 AsylbLG). Children and pregnant asylum seekers are exempt from this regulation and further services can be accessed on a case-by-case basis (Section 6 AsylbLG). Nevertheless, this regulation has been shown to prevent asylum seekers from receiving needs-based care [2, 3]. In addition, language, financial, geographical or structural factors can also act as barriers to accessing adequate healthcare for refugees [4]. Moreover, it is not only access to healthcare but also the circumstances in the host country after migration which are of great relevance to the health of refugees. Factors such as an insecure residency status, satisfaction with the living situation and opportunities for social and economic participation can influence health and well-being [5].

**Info box 1**

In this article, the term ‘refugees’ refers to all people who have applied for asylum at the German Federal Office for Migration and Refugees (BAMF) – regardless of the outcome of their asylum application – as well as people admitted to Germany for resettlement in accordance with the Geneva Refugee Convention of the United Nations High Commissioner for Refugees (UNHCR).

**Info box 2**

The publication by Bozorgmehr et al. [6] defined ‘people with a migrant background’ according to the definition of the International Organisation for Migration (IOM) as ‘a person who moves away from his or her place of usual residence, whether within a country or across an international border, temporarily or permanently, and for a variety of reasons’ [7].

Against this backdrop, population-based data are particularly important in determining healthcare needs. In addition to routine clinical data, data from surveys and interviews at national or regional levels form an essential part of national data systems. Only they can provide reliable information on the frequency of certain diseases as well as potential access barriers. Furthermore, data on residential locations which are used in household surveys can also be used, for example to assess geographical barriers to accessing healthcare. However, a recent analysis of health data available for people with a migrant background (Info box 2) in Europe found that the current utilisation of survey data is insufficient [6]. This is partly due to the fact that this population group – which is considered “hard-to-reach” for research purposes – is often under-represented in population-based studies. In Germany, further problems arise when recruiting refugees for health monitoring surveys. On the one hand, refugees cannot be identified in population registers, as these only record data on nationality and do not provide information on legal status. On the other hand, reporting can be delayed, which is why refugees who have recently arrived and who often live in initial reception or collective accommodation centres are under-represented in population registers. Furthermore, people with a migrant background are regularly excluded from studies if the surveys are exclusively in German.

In Germany, the task of collecting and evaluating information on the health of the population lies with the Robert Koch Institute (RKI), amongst other actors. National data on the health status, access to care services, but also on other relevant indicators such as the health behaviour of children, adolescents and adults living in Germany are regularly collected through several interview and examination surveys within the context of health monitoring at the RKI. Over the past two decades, increased efforts have been made to integrate individuals with a migrant background in the German Health Interview and Examination Survey for Children and Adolescents (KiGGS) and the German Health Interview and Examination Survey for Adults (DEGS1). Such efforts have included oversampling of participants without German nationality, providing multilingual questionnaires and targeted public outreach to recruit people with a migrant background [8]. Since 2016, the RKI has been working more intensively on migration-sensitive recruitment and data collection procedures as part of the Improving Health Monitoring in Migrant Populations (IMIRA) project [8]. However, the samples for these surveys are recruited based on data from the population registration office, which do not adequately represent refugees and asylum seekers in initial reception and collective accommodation centres in Germany [9].

The German Institute for Economic Research’s (DIW) ‘IAB-SOEP-BAMF Panel’, a survey specifically designed to collect information from refugees, is sampled based on the Central Register of Foreign Nationals (AZR). The AZR is kept by the Federal Office for Migration and Refugees (BAMF) as a police register and contains detailed information on the legal status and place of residence of refugees arriving in Germany. Using this as its basis, the IAB-SOEP-BAMF Panel is able to draw a representative sample of refugees in Germany [10]. However, this survey is primarily concerned with socioeconomic aspects such as educational status and the integration of refugees in the labour market. The survey includes questions on general and mental health status [5], but little attention is given to other health-related matters. Questions on utilisation of services are not included, except for a few variables on the uptake of outpatient and inpatient care.

In order to close these gaps in the availability of survey data, a data collection approach was developed as part of the project ‘Improving regional health system responses to the challenges of forced migration’ (RESPOND) in 2016. Funded by the Federal Ministry of Education and Research (BMBF), this project set out to conduct a population-based health survey among refugees in initial reception and collective accommodation centres. This paper presents the project's methodological approach as well as selected results regarding health status, utilisation of healthcare services and quality of care. Furthermore, data on the accommodation situation, the quality of accommodation and the geographical accessibility of primary healthcare are reported.

## 2. Methodology

The present survey was designed as a population-based, cross-sectional study and conducted as part of the RESPOND project. The target population was defined as adult refugees living in initial reception centres (EA) and collective accommodation centres (GU) in the state of Baden-Württemberg at the time of the survey.

### 2.1 Questionnaire development

Drawing on previous feasibility studies [11–13] and using established instruments, a questionnaire was developed that covers essential dimensions of health status, healthcare utilisation, quality of care as well as sociodemographic information and adequately takes into account the specific context and living conditions of refugees. A description of the questionnaire development, including a detailed overview of instruments used, has been published previously [14]. Only a selection of the most important indicators will therefore be presented below.

Health status was assessed using instruments from the European Health Interview Survey (EHIS; general health, pain, chronic diseases) [15] as well as scales for depressive symptoms (PHQ-2; depression) [16] and symptoms of general anxiety disorders (GAD-2) [17]. Both PHQ-2 and GAD-2 scores above a cut-off of three were considered as indicating a depressive or anxiety disorder respectively [16]. Utilisation of healthcare services was assessed based on EHIS instruments (use of specialist and general medical services), the EU Statistics on Income and Living Conditions (EU-SILC; unmet needs) [18] and the German Health Interview and Examination Survey for Adults (DEGS; advice on health behaviour) [19]. Variables of health status, utilisation of healthcare services, quality of care and perceived distance from health services were dichotomised for the analysis (Annex Table 1).

Basic DEGS and EHIS sociodemographic items were supplemented with an adapted version of the MacArthur Scale (subjective social status) [20], as well as questions related to legal status, health insurance status and length of stay in Germany (Annex Table 1). With regard to ‘nationality’ and ‘mother tongue’ variables, only categories that described at least 2% of the participants were considered in the evaluation, remaining answers were categorised as ‘other’. Levels of education were recorded based on the questions of EHIS on school education and vocational qualification and combined in a separate classification into three educational levels. An adapted MacArthur Scale of subjective social status (SSS) in Germany was divided into low SSS (levels 1–4), medium SSS (levels 5–6) and high SSS (levels 7–10) [20, 21].

A number of aspects related to quality of care were examined. On the one hand, ambulatory care sensitive hospitalisations (ASH) were assessed using questions on specific clinical diagnoses and hospitalisations due to these conditions [22]. These are hospitalisations for diseases that are considered potentially avoidable given effective primary care and can therefore be considered as an indicator of the quality of primary care. These are also referred to as ‘avoidable hospitalisations’. In addition, the World Health Organization (WHO) Responsiveness Scale was used to assess non-technical aspects of quality of care in the dimensions of cleanliness, respectful treatment, confidentiality, autonomy in decision-making, communication, choice of provider and waiting time during the last appointment [23]. As the WHO Responsiveness Scale specifically focuses on assessing a patient’s most recent appointment, responses from individuals who had not been to see a doctor were excluded. The questionnaire also included a question on the abuse of medicines from the Structured Clinical Interview for DSM-5 (SCID; medication abuse) [24]. Possible geographical barriers to accessing care were captured using a subjective evaluation of the distance to different care services (pharmacies, primary and specialist care providers, hospitals), taken from the European Patient’s Forum (EPF) study [25].

The questionnaire was developed in English and German and then translated into Albanian, Arabic, Persian, French, Russian, Serbian and Turkish using a TRAPD (Translation, Review, Adjudication, Pretesting and Documentation) approach. Two independent professional translations were brought into a joint discussion, and an interdisciplinary translation and research team was then tasked with the synthesis of both texts [26]. A cognitive pre-test was conducted for several questionnaire items to ensure comprehensibility [27]. The final version of the questionnaire comprised 68 questions.

An instrument was developed to quantify the quality of housing in terms of its structural condition (small-area housing environment deterioration, SHED) and validated in a separate study [28]. Drawing on the Broken Windows Index [29], this instrument measures the condition of (1) window panes and glass, (2) walls and roof, (3) litter, (4) graffiti inside and outside the building, and (5) external spaces on the basis of five observer-based assessments. The instrument has been shown to be highly reliable when conducted in the form of independent individual ratings [28]. In the context of this study, however, it was used as a rating by a team, as the joint work on site did not create an independent, but a combined impression of the residential environment. A sixth question assessed the general living environment as a global rating. Following Z-standardisation and 0–1 normalisation of the individual results for the purpose of comparability, the variables collected on the quality of accommodation were converted into an overall score. Facilities were divided into quintiles based on the overall score in order to examine accommodation quality based on the distribution of people living in the centres.

### 2.2 Sampling

This study had no access to the AZR data so a separate sampling frame was constructed. Sampling was carried out at the level of accommodation centres. After arrival and registration by the BAMF, refugees are accommodated in initial reception centres of the federal states. At the point of data collection, refugees were allowed to stay in these centres for a maximum of six months, with the exception of persons from so-called ‘safe countries of origin’ (Section 47 Asylum Act, AsylG). Refugees with good prospects of being allowed to stay in the country may then be transferred to collective accommodation centres at regional level. In the initial reception centres, the reception authorities at the federal state level are responsible for accommodation; the responsibility for refugees in collective accommodation centres and follow-up accommodation lies with the regional and district authorities.

A list of all initial reception centres in the state as well as anonymised occupancy lists at the room level was established in co-operation with the Ministry of the Interior of Baden-Württemberg and the responsible regional councils. A two-stage random sample was drawn from a total of twelve centres. In the first stage, six of the twelve centres were selected with a probability proportional to accommodation occupancy and responsible authority. In the second stage, a random selection was made at room level so that 25% of the residents were included in the sample. This self-weighting approach results in an equal selection probability for each person within the sampled population.

The sampling procedure for collective accommodation centres has been described in detail previously [14]. All lower-level reception authorities were contacted in order to obtain a list of all collective accommodation centres (N = 1,933), as well as the corresponding occupancy figures, of the 44 districts of Baden-Württemberg. This was done in cooperation with the Ministry of Social Affairs and with the consent of the County Association (Landkreistag) of Baden-Württemberg. At the time of the survey, a total of 70,634 refugees were living in collective accommodation centres. A random sample proportional to the population was drawn at the level of accommodation centres, balancing on the number of refugees in the district as well as accommodation size. A total of 65 centres were drawn to include a net sample of 1% of all refugees at district level.

An additional benefit of manually collating the sampling frame at the level of collective accommodation centres was the possibility of identifying geographical locations. The geo-coordinates of 1,786 centres were determined. As some authorities did not provide geo-information, 7.6% (n = 147) of centres from five urban and rural districts were excluded from geographical analysis because their addresses could not be determined.

### 2.3 Study implementation

Specifically trained, multilingual research staff collected the data between February and June 2018. Refugees living in the centres were contacted at least one week in advance by the staff or responsible social workers at the centre to inform about the purpose and time of the visit. In order to reach a large proportion of the residents, each centre was visited on two consecutive days. In the course of field visits, the research staff completed questionnaires on accommodation quality for each accommodation centre in the sample.

All people living in sampled facilities were personally informed about the study by multilingual field teams on site and invited to participate (‘door-to-door recruitment’ [30]). Standardised, multilingual audio messages were also used. Criteria for inclusion in the study were being at least 18 years old and proficiency in at least one of the nine study languages. Illiterate people were included in the study if they confirmed that someone could help them fill out the questionnaire. Potential participants received a questionnaire and a leaflet with study information in one of the nine languages, as well as non-monetary, unconditional incentives (notebooks, pens and colouring pads/crayons for children). Respondents could choose between returning the completed questionnaire in person to the research team or, alternatively, returning it by post in a pre-paid envelope. In addition, an online version of the questionnaire (using a personalised QR code) was also made available. If people were approached who could not participate in the study or did not meet the inclusion criteria, the reason for non-participation, their gender and language were documented.

### 2.4 Weighting

The RESPOND data was weighted to improve the accuracy of the sample when making estimates regarding the total refugee population. The weights were calculated using data on gender, age group and region of origin from Baden-Württemberg’s asylum statistics [31]. For country of origin, data on asylum applications from 2016 to 2018 (quarters 1 to 4) were available. For gender and age group, statistics were only available for one quarter each of 2016 (Q2), 2017 (Q4) and 2018 (Q3). These asylum application statistics can only approximate the true composition of the refugee population, as first-time applicants before 2016 as well as applicants that apply for asylum more than once are generally not recorded. To enable weighting with a complete data matrix, missing values were imputed using the ‘mice’ package in R [32]. The complete data matrix was then used to calculate calibration weights. Data on gender, age and region of origin were adjusted to the distribution of these variables in the asylum statistics, taking into account the sample design and using ‘iterative proportional fitting’ (raking technique) [33].

### 2.5 Data evaluation

Descriptive statistics of the weighted data are used to determine physical and mental health status, utilisation of health services, unmet needs, quality of care as well as the perceived geographical distance to healthcare services. For this purpose, prevalence of each indicator, including 95% confidence intervals, are presented by gender (health status and utilisation) or by type of accommodation (responsiveness and perceived geographical distance). These analyses were carried out with STATA version 15.1.

To calculate the distance to primary care services, geo-data on general medical practices from the publicly available database of the Association of Statutory Health Insurance Physicians (Kassenärztliche Vereinigung) of Baden-Württemberg were used. Geo-information software (QGIS) was used to determine the nearest practice, which was then assigned for each centre based on linear distance and using the ‘nearest neighbour analysis’. As refugees usually do not have their own car, calculating travel time by public transport or on foot is particularly important. The travel times (walking, driving and public transport) were calculated using the Google Maps Distance Matrix API (last calculation: 19 June 2020, 07:00) [34]. Google Maps’ Distance Matrix API offers the advantage of simultaneous requests for several data points. Travel time and date were randomly selected for a working day.

## 3. Results

**Annex Figure 1:**
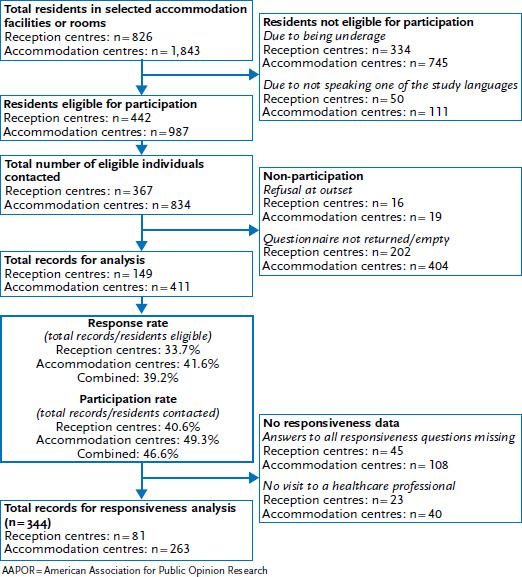
Response rate calculated according to AAPOR criteria Source: RESPOND Study 2018

A total of 560 adult refugees (response rate 39.2%; Annex Figure 1) took part in the study, of which 411 (73.4%) of which lived in collective accommodation centres, with the remaining 149 (26.6%) living in initial reception centres. The response rate was calculated according to the recommendations of the American Association for Public Opinion Research (AAPOR) [35]. Almost one third (n = 158; 31.3%) of the sample were women, more than half (n = 253; 51.4%) were under 31 years of age. The primary regions of origin were West Asia (n = 134; 26.7%), South Asia (n = 128; 25.5%) and West Africa (n = 120; 23.9%). Educational status was mixed, but the subjective social status in Germany was predominantly (n = 277; 70.7%) assessed as being low. More than half of participants had already been in Germany for more than one year (n = 253; 55.8%), but the majority (n = 281; 62.2%) still had asylum seeker status. In initial reception centres, there was a tendency toward shorter length of stay in Germany and a more uncertain asylum status. Half of the participants (n = 240; 52.2%) held an electronic health card (Annex Table 2).

### 3.1 Health status

**Figure 1:**
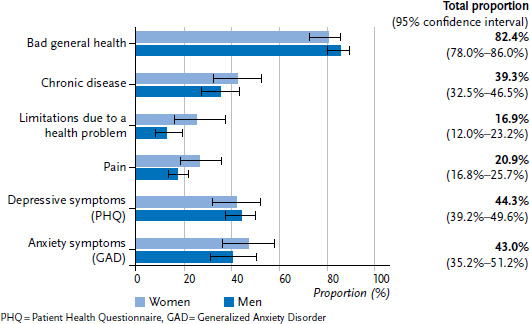
Self-reported, weighted prevalence of health issues and symptoms by gender (with 95% confidence intervals) Source: RESPOND Study 2018

After weighting the data, 82.5% of refugees reported either a moderate, poor or very poor general health status. In addition, 39.3% of respondents reported a chronic illness, 16.9% a limitation due to a health problem and 20.9% suffered from severe to very severe pain. There was a tendency towards a higher prevalence of health limitations as well as pain among female refugees (Figure 1). The prevalence of depressive symptoms was 44.3%, and 43.0% for symptoms of anxiety (Figure 1).

### 3.2 Utilisation of healthcare services

**Figure 2:**
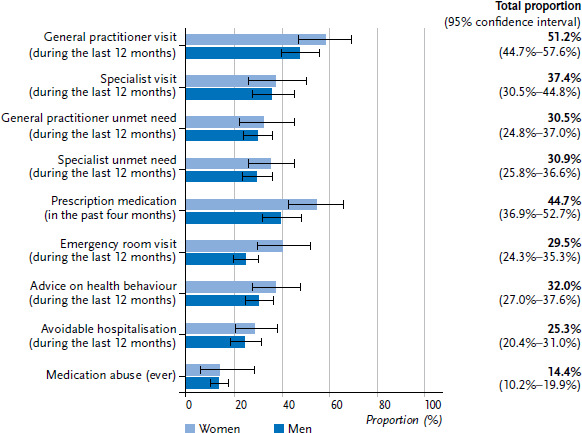
Self-reported, weighted utilisation and quality of health services by gender (with 95% confidence intervals) Source: RESPOND Study 2018

In the twelve months prior to the survey, 51.2% of refugees had visited primary and 37.4% specialist care services. Almost one third of refugees reported unmet needs (foregone health services), both in primary and specialist care. 29.5% of refugees had made use of emergency care in the past twelve months, whereas just under half had received prescription medication during the four weeks prior to the study. For both emergency care and prescription medication, there was a clear trend towards a greater utilisation by female refugees. One third of respondents had received advice from their doctor regarding their health behaviour in the twelve months prior to the study (Figure 2).

### 3.3 Quality of care

**Figure 3:**
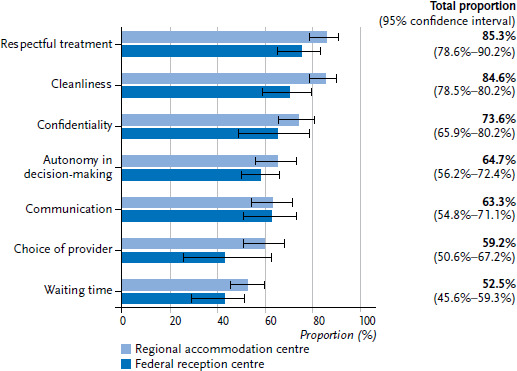
Quality of care perceived as good or very good (responsiveness) by type of accommodation (weighted, with 95% confidence intervals) Source: RESPOND Study 2018

One quarter of refugees stated having been in inpatient treatment in the twelve months prior to the survey due to medical conditions which, with adequate primary care, should not have required hospitalisation (avoidable hospitalisations). In addition, 14.4% of respondents reported having been addicted to prescription drugs or having taken more of a drug than they had been prescribed at least once in their life. Reported responsiveness of care varied by type of healthcare service and accommodation type (collective accommodation/initial reception centre; Figure 3). The best ratings were given for respectful treatment and cleanliness, while choice of provider and waiting time received the worst ratings. When compared to the initial reception centre setting, there was a tendency towards a subjectively better assessment of care services for respondents in collective accommodation across all responsiveness domains; this tendency was particularly clear for cleanliness (Figure 3).

### 3.4 Quality of accommodation

In total, the 560 respondents were accommodated in 63 different centres. The quality of accommodation of 61 of them was assessed and calculated, and covered five initial reception centres and 56 collective accommodation centres. With a possible spectrum from very high (value = o) to very low (value = 6) accommodation quality, collective accommodation received a better average rating of 1.0 (median = 0.5; min. 0.0; max. 4.8) than initial reception centres with an average of 2.7 (median = 1.7; min. 0.5; max. 5.2). However, when the accommodation size is taken into account, 45.3% of refugees lived in three accommodation centres that all received very low ratings for accommodation quality (lowest quintile) (one initial reception centre, two collective accommodation centres) (Table 1).

### 3.5 Geographical distance to healthcare services

**Figure 4:**
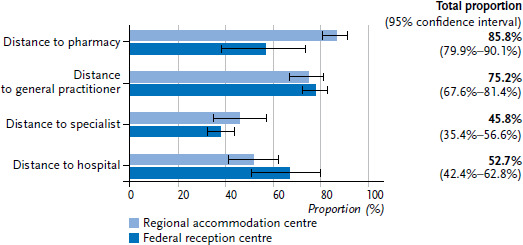
Distance to pharmacies, general practitioners, specialists and hospitals perceived as ‘close enough’ by type of accommodation (weighted, with 95% confidence intervals) Source: RESPOND Study 2018

85.8% of refugees stated that a pharmacy was close enough to their accommodation. 75.2% said that primary medical services were close enough, while the same was true of only 45.8% for a specialist practice and 52.7% for a hospital. Pharmacies tended to be judged as being ‘close enough’ more frequently by refugees in collective accommodation centres, while hospitals were judged as ‘close enough’ more frequently by refugees in reception centres (Figure 4).

**Figure 5:**
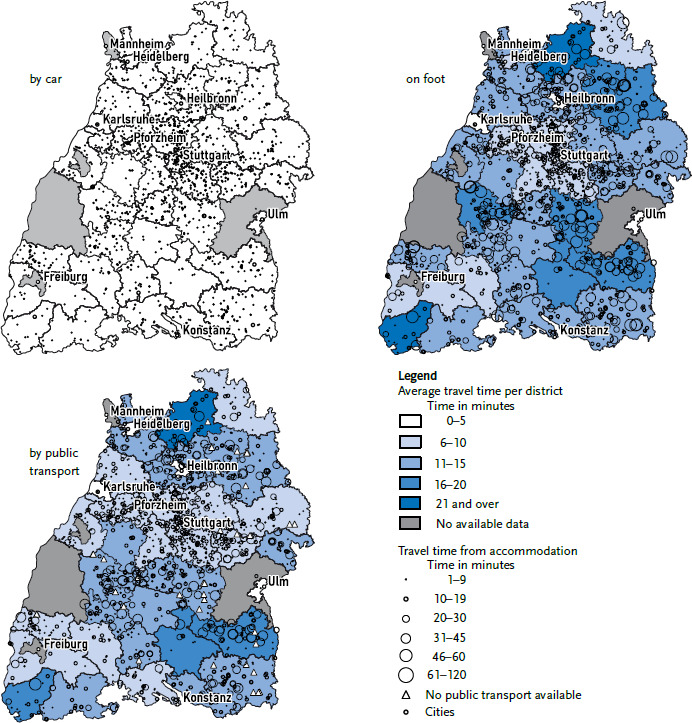
Travel time (in minutes) to the nearest primary care practice per accommodation and mean travel time per district by car, on foot and by local public transport Source: RESPOND Study 2018

Figure 5 shows the actual distances from all collective accommodation centres in Baden-Württemberg to the nearest primary care practice. The mean travel time by car was 2.7 minutes (standard deviation 2.1; min. 0; max. 18.7). All collective accommodation centres were within 30 minutes of the nearest practice by car (Figure 5); only about 90% of the centres had a practice within 30 minutes walking distance (Figure 5). The mean walking time was 13.2 minutes (standard deviation 15.5; min. 0; max. 119.3).

91% of accommodation centres had a practice within a 30-minute journey by public transport (Figure 5). The average travel time by public transport was 11 minutes (standard deviation 11.03; min. 0; max. 97.08), yet 41 accommodation centres were not connected to the public transport network. For these 41 accommodation centres, the travel time on foot was at least 60 minutes, and the walking distances were between 4.5 and 10 kilometres each way. The travel time by car from these accommodations to the nearest primary care practices was nine minutes on average (standard deviation 2.8 min. 4.2; max. 18.7), with locations ranging from five to just under 16 kilometres away. In addition to the 41 accommodation centres mentioned, another 40 accommodation centres had more than 45 minutes travel time from the respective nearest practice, both on foot and by public transport.

## 4. Discussion

The RESPOND study is characterised by its population-based sampling procedure, multilingual questionnaires based on established instruments and personal contact with respondents, relevant authorities and institutions. This made it possible to obtain reliable epidemiological data on the health status, access to and quality of healthcare as well as important aspects of the living and housing environments of refugees. In general, refugees have a high overall health burden. For example, 44.3% report depressive symptoms, a very high figure compared to the general population in Germany (10.1%) [36], which points to a high need for health and psychosocial services. In other areas, such as limitations in everyday life due to a health problem, the figures for refugees (16.9%) are also higher than for the general German population (6.6%) [37]. Direct comparisons are difficult because of the differences in age and gender composition between the two populations. Important insights can nonetheless be gained from such comparisons, which should be improved through the use of population standardisation in future studies.

The high mental health burden of refugees in Germany has been shown previously by analyses based on the IAB-SOEP-BAMF panel [5, 38]. However, when considering the burden of physical illnesses, the two studies come to different conclusions: compared to the population living in Germany, the IAB-SOEP-BAMF panel [38] records a lower burden, while the RESPOND study shows a higher burden. To a certain extent, this can be explained by the fact that the RESPOND study mainly captures recently arrived refugees (since 2016), whereas the IAB-SOEP-BAMF panel analyses were based on a sample of refugees which arrived in Germany between 2013 and 2016. In addition, RESPOND is the first study which facilitated population-based insights on utilisation, accessibility and quality of care for refugees – topics not covered by the IAB-SOEP-BAMF panel.

The majority of refugees had used healthcare services in the twelve months prior to the survey. However, a high number of respondents reported foregone care. The comparatively high prevalence of avoidable hospitalisations also points to an insufficient coverage of primary care services. With regard to the quality of care experienced (responsiveness), the overall assessment of cleanliness and respectful treatment were good, but assessments of choice of provider and waiting time showed room for improvement. Compared to a study of patients with chronic illnesses in outpatient care in Germany [39], refugees in the RESPOND study rated every domain of responsiveness as worse. A close analysis of the responsiveness of the healthcare system for refugees, including a qualitative analysis of the possible reasons for differences between the different domains from the perspective of those affected, is urgently needed to comprehensively assess how refugees experience the quality of care.

Important insights were also gained with regard to the quality of accommodation facilities. While the majority of centres visited were in good or acceptable structural condition, a disproportionately large number of refugees were living in large accommodation centres which were in poor condition. Findings from existing research in Germany shows that structurally poor housing conditions can negatively impact refugee’s mental health [40]. In addition, the international literature points to links between the quality of accommodation, occupancy density and physical health, particularly in relation to the worsening of chronic diseases such as asthma and the spread of infectious diseases [41].

The COVID-19 pandemic has made explicit the importance of the link between the housing conditions of refugees and their health: in centres with better conditions and lower occupancy levels, authorities had better opportunities to comply with physical distancing, isolation and quarantine requirements, thereby being more effective in controlling the pandemic [42]. The implementation of existing standards for the accommodation of refugees should be re-examined with respect to the structural quality of buildings, occupancy density, geographic location and cleanliness. In addition, further research on the impact of different housing and living conditions on the health of refugees, including accommodation quality, is needed to support the planning of accommodation processes from a health perspective. In this context, qualitative research is also of great importance in providing insights to the significance of the ‘living environment’ from the perspective of refugees and in shedding light on the connections between the living environment and health in the unique context of collective accommodation facilities.

Primary care services are easily accessible from collective accommodation facilities by car, on foot or by public transport for most refugees. The average distance travelled by car was less than the ten minutes generally reported for the German population [43] for all included districts. However, access to selected centres proved difficult, especially in rural areas. The question therefore arises as to whether it makes sense to accommodate refugees, who often do not have a car, in structurally underdeveloped regions. This study benefited from the Google Maps Distance Matrix API, which enabled the analysis of travel times by public transport. However, the analysis was limited to one practice and a single time of travel. Further analyses should aim to extend this to multiple primary care practices, other healthcare services and travel times at different points of the day.

This is the first population-based study in Germany that goes beyond individual diseases to map the health situation of refugees in collective accommodation facilities in a German federal state. In comparison to other population-based surveys of the general population, a high response rate was achieved. The approach shows that migration-sensitive health monitoring for refugees in initial reception and collective accommodation centres is possible in principle and can complement existing approaches to recruiting refugees via population registers. Refugees are not per se difficult to reach within the context of empirical surveys, although other approaches are necessary in addition to those usually used in Germany to date. The study was limited by the fact that it was restricted to one federal state and worked with a relatively small sample size. However, the instruments and the sampling method applied by the RESPOND survey have already been successfully repeated in Berlin [44]. Expanding the approach to other federal states and giving continuity to the described approaches can improve the empirical foundation of healthcare provision for refugees and close existing gaps in health monitoring.

## Key statements

- To date, the integration of refugees in German health surveys is insufficient.
- The results of the RESPOND study indicate a high health burden, while at the same time showing high unmet needs.
- Primary care services are accessible geographically, but quality indicators suggest other access barriers.
- Almost half of all refugees (45.3%) live in accommodation facilities of poor structural quality.
- The collection of valid data on the health of refugees should be continued and extended to other federal states.

## Figures and Tables

**Table 1:** Number of regional accommodation centres (GU) and federal reception facilities (EA) according to accommodation quality in quintiles as well as their respective number of residents Source: RESPOND Study 2018

Quality of accommodation in quintiles	GU (n = 56)		EA(n = 5)		Total (n = 61)		Residents (n = 5,092)	
	Number	%	Number	%	Number	%	Number	%
Q1 (very high)	40	71.4	1	20	41	67.2	1,423	27.9
Q2 (high)	12	21.4	2	40	14	23.0	1,297	25.5
Q3 (average)	2	3.6	0	0	2	3.3	26	0.5
Q4 (low)	1	1.8	0	0	1	1.6	41	0.8
Q5 (very low)	1	1.8	2	40	3	4.9	2,305	45.3
Total	56	100.0	5	100.0	61	100.0	5,092	100.0

Q = quintile, GU = regional accommodation centre, EA = federal reception centre

## Appendix

**Annex Table 1:** Selected variables of the RESPOND questionnaire, their source and categorisation Source: RESPOND Study 2018

Variable	Source	Categorisation
**Sociodemographic data**		
Age	DEGS	18–25, 26–30, 31–35, 36–40, ≥41 years old
Gender	DEGS	1 = male 2 = female
Nationality	DEGS	Region of origin according to UN Geoscheme
Educational status	EHIS	School and professional education
Months since arrival in Germany	–	0–6, 6–12, 13–15, 16–24, 24–36 months
Legal status	–	1 = Asylum seeker 2 = Refugee status awarded 3 = Refugee status rejected/temporary suspension of deportation
Health insurance card	–	1 = yes 0 = no
Subjective social status in Germany	MacArthur Scale	1 = low SSS 2 = medium SSS 3 = high SSS
**Health status**		
General health status	EHIS	1 = moderate to very poor condition 0 = good/very good condition
Chronic diseases	EHIS	1 = present 0 = not present
Health limitations	EHIS	1 = severe limitations 0 = moderate/no limitations
Pain	DEGS	1 = severe/very severe pain 0 = moderate to no pain
Depressive symptoms	Patient Health Questionnaire, 2-item version	1 = PHQ-2 value ≥3 0 = PHQ-2 value <3
Anxiety symptoms	Generalized Anxiety Disorder, 2-item Version	1 = GAD-2 value ≥3 0 = GAD-2 value <3
**Use of health services**		
Primary care visit	EHIS	1 = Primary care visit <12 months 0 = Primary care visit >12 months/never
Specialist visit	EHIS	1 = Specialist visit <12 months 0 = Specialist visit >12 months/never
Unmet need for primary care	EU-SILC	1 = unmet needs 0 = no unmet needs
Unmet need for specialist	EU-SILC	1 = unmet needs 0 = no unmet needs
Prescription medicines	EHIS	1 = Medication was prescribed 0 = no medication was prescribed
Emergency room visit	EHIS	1 = Emergency room visit <12 months 0 = Emergency room visit >12 months
Health behaviour advice	DEGS	1 = Health behaviour advice 0 = no health behaviour advice
**Quality of care**		
Ambulatory care sensitive hospitalisations	EHIS	1 = Hospitalisation due to ASC 0 = no hospitalisation due to ASC
Medication abuse	SCID	1 = Medication abuse 0 = no medication abuse
Responsiveness: respectful treatment	WHS	1 = good/very good responsiveness 0 = moderate to very poor responsiveness
Responsiveness: Cleanliness	WHS	1 = good/very good responsiveness 0 = moderate to very poor responsiveness
Responsiveness: Confidentiality	WHS	1 = good/very good responsiveness 0 = moderate to very poor responsiveness
Responsiveness: Autonomy in decision-making	WHS	1 = good/very good responsiveness 0 = moderate to very poor responsiveness
Responsiveness: Communication	WHS	1 = good/very good responsiveness 0 = moderate to very poor responsiveness
Responsiveness: Choice of provider	WHS	1 = good/very good responsiveness 0 = moderate to very poor responsiveness
Responsiveness: Waiting time	WHS	1 = good/very good responsiveness 0 = moderate to very poor responsiveness
**Distance of supply**		
Perceived distance pharmacy	EPF Access to Healthcare	1 = close enough 0 = not close enough
Perceived distance General practitioner	EPF Access to Healthcare	1 = close enough 0 = not close enough
Perceived distance of specialist	EPF Access to Healthcare	1 = close enough 0 = not close enough
Perceived distance hospital	EPF Access to Healthcare	1 = close enough 0 = not close enough

EHIS = European Health Interview Survey, UN = United Nations, SSS = subjective social status, DEGS = German Health Interview and Examination Survey for Adults,

PHQ-2 = Patient Health Questionnaire 2-item version, GAD-2 = General Anxiety Disorder 2-item version, EU-SILC = EU Statistics on Income and Living Conditions,

SCID = Structured Clinical Interview for DSM-5, ASC = ambulatory-sensitive conditions, WHS = World Health Survey, EPF = European Patient’s Forum

**Annex Table 2:** Sociodemographic characteristics of the study participants by type of accommodation Source: RESPOND Study 2018

	GU (n = 411)		EA (n = 149)		Total (n = 560)	
	Number	%	Number	%	Number	%
**Age group in years**						
18–25 years	117	32.5	47	35.6	164	33.3
26–30 years	60	16.7	29	22.0	89	18.1
31–35 years	62	17.2	25	18.9	87	17.7
36–0 years	52	14.4	14	10.6	66	13.4
≥41 years	69	19.2	17	12.9	86	17.5
Total	360	100.0	132	100.0	492	100.0
**Sex**						
Male	115	31.3	43	31.2	158	31.3
Female	252	68.7	95	68.8	347	68.7
Total	367	100.0	138	100.0	505	100.0
**Region of origin**						
Eastern Europe	12	3.2	0	0.0	12	2.4
Southern Europe	6	1.6	12	9.2	18	3.6
West Asia	112	30.2	22	16.8	134	26.7
South Asia	119	32.1	9	6.9	128	25.5
West Africa	63	17.0	57	43.5	120	23.9
Central Africa	9	2.4	5	3.8	14	2.8
North Africa	2	0.5	1	0.8	3	0.6
Other nationalities	48	12.9	25	19.1	73	14.5
Total	371	100.0	131	100.0	502	100.0
**Educational status**						
Low	102	35.9	27	24.5	129	32.7
Medium	122	43.0	51	46.4	173	43.9
High	60	21.1	32	29.1	92	23.4
Total	284	100.0	110	100.0	394	100.0
**Months since arrival in Germany**						
0–6 months	53	15.5	94	81.0	147	32.0
6–12 months	39	11.4	17	14.7	56	12.2
13–15 months	95	27.7	4	3.4	99	21.6
16–24 months	130	37.9	0	0.0	130	28.3
24–36 months	26	7.6	1	0.9	27	5.9
Total	343	100.0	116	100.0	459	100.0
**Legal status**						
Asylum seeker	177	54.3	104	82.5	281	62.2
Asylum approved	76	23.3	3	2.4	79	17.5
Refusal/temporary suspension of deportation	73	22.4	19	15.1	92	20.4
Total	326	100.0	126	100.0	452	100.0
**Electronic Health Card**						
No	123	35.2	94	87.0	217	47.5
Yes	226	64.8	14	13.0	240	52.5
Total	349	100.0	108	100.0	457	100.0
**Subjective social status in Germany**						
Low	200	69.9	77	72.6	277	70.7
Medium	57	19.9	13	12.3	70	17.9
High	29	10.1	16	15.1	45	11.5
Total	286	100.0	106	100.0	392	100.0

GU = regional accommodation centres,

EA = federal reception centre
